# MAN2B1 in immune system-related diseases, neurodegenerative disorders and cancers: functions beyond α-mannosidosis

**DOI:** 10.1017/erm.2024.34

**Published:** 2024-12-04

**Authors:** Yuwen Han, Yuanshuai Zhou, Jinlin Pan, Minxuan Sun, Jiao Yang

**Affiliations:** 1 School of Biomedical Engineering (Suzhou), Division of Life Sciences and Sciences and Medicine, University of Science and Technology of China, Hefei 230026, China; 2 Jiangsu Key Lab of Medical Optics, Suzhou Institute of Biomedical Engineering and Technology, Chinese Academy of Sciences, Keling Road No.88, Suzhou 215163, China; 3 Institute of Clinical Medicine Research, Suzhou Hospital, Affiliated Hospital of Medical School, Nanjing University, Lijiang Road No. 1, Suzhou 215153, China; 4 Suzhou Research Center of Medical School, Suzhou Hospital, Affiliated Hospital of Medical School, Nanjing University, Lijiang Road No. 1, Suzhou 215153, China

**Keywords:** α-mannosidosis, cancer, immunity, MAN2B1, neurodegenerative disorders

## Abstract

Glycosylation modifications of proteins and glycan hydrolysis are critical for protein function in biological processes. Aberrations in glycosylation enzymes are linked to lysosomal storage disorders (LSDs), immune interactions, congenital disorders and tumour progression. Mannosidase alpha class 2B member 1 (MAN2B1) is a lysosomal hydrolase from the α-mannosidase family. Dysfunction of MAN2B1 has been implicated as causative factors in mannosidosis, a lysosomal storage disorder characterised by cognitive impairment, hearing loss and immune system and skeletal anomalies. Despite decades of research, its role in pathogenic infections, autoimmune conditions, cancers and neurodegenerative pathologies is highly ambiguous. Future studies are required to shed more light on the intricate functioning of MAN2B1. To this end, we review the biological functions, expression patterns, enzymatic roles and potential implications of MAN2B1 across various cell types and disease contexts. Additionally, the novel insights presented in this review may aid in understanding the role of MAN2B1 in immune cells, thereby paving the way for targeted therapeutic interventions in immune-related disorders.

## Introduction

The glycosylation modification of proteins and the turnover processes of glycan hydrolysis play a pivotal role in maintaining essential protein functions across various biological processes [1, 2]. Aberrations in enzymes involved in glycosylation have been linked to lysosomal storage disorders (LSDs), immune intercellular receptor–ligand interactions, congenital disorders and tumour progression [3-9]. Mannosidosis, characterised by mental retardation, hearing impairment and anomalies in immune function and skeletal development, is a lysosomal storage disease stemming from an inherited deficiency in the lysosomal enzyme α-mannosidase [10, 11].

Lysosomal α-mannosidase, encoded by the MAN2B1 (LAMAN) gene, belongs to the α-mannosidase II family, catalysing the hydrolysis of terminal non-reducing **α**-D-mannose residues within α-D-mannosides during the ordered degradation of N-linked glycans [12]. Deficiency in MAN2B1 leads to impaired lysosomal function and compromised glycoprotein degradation, resulting in the gradual accumulation of mannose-rich oligosaccharides in cells and tissues, culminating in **α**-lysosomal storage disease [13, 14]. α-Mannosidosis is a progressive multisystemic disease [15]. In the past, α-mannosidosis was classified into three phenotypic subtypes, with type 1 designated as the mild form and type 3 as the severe form (Table 1).The majority of infants with α-mannosidosis are born without any apparent abnormalities. However, the disease becomes clinically apparent during infancy and childhood, manifesting as delayed psychomotor development, hearing loss, intellectual disability, skeletal dysmorphia and neurological deterioration affecting mobility [16]. Engin Köse conducted a long-term clinical evaluation of patients with α-mannosidosis, examining demographic, clinical, laboratory and molecular characteristics. The evaluation found that the early initiation of enzyme replacement therapy (ERT) may lead to a better clinical outcome [17].

**Table 1.** Three phenotypic types of α-mannosidosis [3, 16, 18]

Phenotypic type	Type I	Type II	Type III
Severity	Less severe	Moderate	Severe
Age of onset	>10 years	≤10 years	Early infancy
Progression	Extremely slow	Slow	Rapid
Clinical symptoms	Hearing loss, ataxia, psychiatric disorder, skeletal disorder, intellectual disability	Speech delay, hearing loss, developmental delay, motor disturbances/joint laxity, characteristic facial features, infections, mild hepatosplenomegaly, hernia	Skeletal abnormalities, facial dysmorphia, profound hearing loss, hepatosplenomegaly, marked and progressive deterioration in motor and cognitive function

Additionally, abnormal expression of MAN2B1 has been associated with various other diseases, encompassing congenital anomalous erythropoietic anaemia type II [19], cancers [20], infectious diseases [21], autoimmune diseases [22] and neurodegenerative conditions [23]. The link between MAN2B1 and mannosidosis has been reviewed elsewhere [3, 24]. In this review, we focus on the expression and activity of MAN2B1 and its correlation with diverse diseases, aiming to provide therapeutic targeting insights for both researchers and clinicians.

## Biological function of MAN2B1

Based on biochemical properties, catalytic mechanisms and conserved amino acid sequences within characteristic regions, α-mannosidases are classified into three categories: class I, class II and unclassified α-mannosidases. Class I α-mannosidases exhibit a molecular mass ranging from 63 to 73 kDa and possess a conserved sequence belonging to the glycoside hydrolase 47 family. Predominantly located in the endoplasmic reticulum, Golgi apparatus and certain cell membrane regions, they play a role in N-glycan secretion and protein surveillance [25, 26]. Class II α-mannosidases have a molecular mass between 107 and 136 kDa and feature conserved sequences aligned with the glycoside hydrolase 38 family. These enzymes are primarily distributed across the endoplasmic reticulum, Golgi, lysosomes and cytoplasm. They are involved in the synthesis and degradation of glycoproteins. Key members of this family include MAN2A1, MAN2A2, MAN2B1, MAN2B2 and MAN2C1 [27]. Recent research has revealed that α-mannosidase is present in not only the glycoside hydrolase 38 and 47 families but also in the glycoside hydrolase 92, 99 and 125 families. These newly discovered α-mannosidases function similarly to class I or class II α-mannosidases, primarily catalysing oligosaccharide reactions [28, 29].

The human lysosomal gene MAN2B1, also referred to as the LAMAN gene or MANB gene, resides on chromosome 19, encompassing a genomic DNA span of 21.5 kb across 24 exons, housing a 2964 bp reading frame [3]. Notably, three primary transcription initiation sites have been identified: −309, −196 and − 191 bp upstream of the ATG initiation sites, respectively [30]. The 134 bp sequence, devoid of the characteristic CAAT or TATA sequence upstream of the transcription start site, contains multiple GC-rich sites within its 5′-end region, facilitating binding to transcription factors such as SP-1, AP-2 and ETF. Upon introduction of the MAN2B1 gene’s 5′ end region into the bacterial CAT gene, it was discovered that the 150 bp sequence at the 5′ end efficiently promotes the expression of the MAN2B1 gene in COS-7 cells (African green monkey SV 40 transformed kidney cells). Furthermore, Gotoda et al. conducted an analysis of the mRNA’s 5′ end region of the human MAN2B1 gene, pinpointing the transcription site at −28 and − 20 bp distant from the start codon ‘ATG’ [31, 32].

The initial transcription product of MAN2B1 is approximately 3.5 kb and encodes a precursor consisting of 988 or 1011 amino acids, mainly due to the start site where translation can be altered. Post-translational modifications of MAN2B1 polypeptides occur within the endoplasmic reticulum. Initially synthesised as a single-stranded precursor, it undergoes processing and cleavage into fragments of 70.42 kDa (abc), 42 kDa (d) and 15 kDa (e) during maturation and subsequent endosomal transport to the lysosome [31, 33]. The 70.42-kDa polypeptides undergo further protease-mediated hydrolysis, forming three peptides interconnected by disulfide bonds, culminating in the assembly of a protein composed of five polypeptides. Ectopic expression of MAN2B1 in COS and CHO cells revealed the secretion of an unprocessed 120-kDa precursor. In its native state, MAN2B1 exists as a homodimer, its enzymatic activity contingent upon zinc ions while being susceptible to inhibition by copper ions [34, 35].

The X-ray crystallographic structure of bovine MAN2B1 is known at a 2.7-A resolution [34]. Within the bovine MAN2B1 structure, four disulfide bridges are established between cysteines C55-C358, C268-C273, C412-C472 and C493-C501, all of which remain conserved in the human MAN2B1 sequence. Utilising the bovine crystal structure as a template, a structural model of the homologous human MAN2B1 was developed in 2011, exhibiting a sequence similarity of up to 84% to the template structure [31, 34, 36]. Human MAN2B1 is characterised by 11 glycosylation sites, where eight of these sites align with the conserved positions identified in the bovine MAN2B1. The MAN2B1 structure comprises a seven-stranded active site a/b domain interconnected by a three-helix bundle to three b-domains. Its optimal pH range spans from 4.5 to 5, displaying only half of the maximum activity at pH levels of 7 and above, under physiological cytosolic and extracellular conditions. Additionally, in vitro assessments have determined the temperature range of 35–40 °C as optimal for maximum enzyme activity, aligning with the physiological temperature range of the human body [37].

MAN2B1 plays a pivotal role in the breakdown of N-linked glycan chains released during the intracellular metabolism of glycoproteins. Primarily operating within acidic lysosomes, MAN2B1 contributes significantly to the orderly degradation of lysosomal N-linked oligosaccharides. It exhibits specificity for terminal non-reducing **α**-1,2, **α**-1,3 and **α**-1,6 mannosidic linkages, particularly in high-mannose and heterozygous N-linked glycans [31, 38]. Northern blotting revealed heightened expression of MAN2B1 in several tissues, notably in the lung, kidney, pancreas and peripheral blood leukocytes. In the central nervous system (CNS), its expression appears notably elevated in the corpus callosum and spinal cord. However, in comparison, expression levels are comparatively lower in regions such as the cerebellum, cerebral cortex, frontal and temporal lobes [39, 40].

Reported mutations within the MAN2B1 gene encompass a spectrum of alterations, including deletions, insertions, duplications, missense, nonsense and splice mutations. Among these, missense mutations stand out as the most prevalent variants [13, 31, 33]. Missense mutations in MAN2B1 can be classified into two primary categories: Active site-disrupting mutations: These genetic alterations result in the production of translationally defective proteins. Although these proteins undergo synthesis, processing and lysosomal translocation, they fail to execute their typical enzymatic functions due to disruptions within their active sites. Misfolded mutations: These mutations lead to the synthesis of proteins with structural misfolding, preventing them from attaining the correct folded configuration. Consequently, these proteins are retained within the endoplasmic reticulum (ER). According to the Human Gene Mutation Database (http://www.hgmd.cf.ac.uk/ac/all.php), a total of 151 mutations of MAN2B1 have been reported, comprising 57 missense mutations, 28 nonsense mutations, 22 splice-site mutations, 19 small deletions, 20 small insertions and 5 gross insertions/duplications. Among these mutations, 143 are associated with α-mannosidosis, 2 with abnormalities of the cardiovascular system, and a few others are related to autism spectrum disorder, developmental disorder and intellectual disability. The missense mutation c.2248C>T (p.Arg750Trp) stands out as a common occurrence among α-mannosidosis patients, having been reported in the majority of European populations studied, comprising over 30% of all detected disease alleles [13].

## MAN2B1 and immune system-related diseases

Recurrent infections represent a predominant clinical feature observed in α-mannosidosis cases, as documented in prior literature [10]. Patients afflicted with α-mannosidosis typically exhibit immunocompromised states, characterised by impaired leukocyte chemotaxis, diminished phagocytic capabilities and disruptions within the IL-2 signalling pathway [41]. This raises a pivotal query regarding the potential involvement of MAN2B1 in modulating immune system functionality. With the burgeoning availability of sequencing data for inflammatory disorders, facilitated by advancements in RNA sequencing technologies and database proliferation, our investigation revealed instances of aberrant MAN2B1 expression or enzymatic activity across various inflammatory diseases, animal models and cellular models (Table 2).

**Table 2.** Implication of MAN2B1 in diverse diseases

Disease type/inducer	Species	Expression	Αctivity	Samples/cell types	Type of study	Reference
Immune system-related diseases							
SLE	Human	Mut	Decreased	Kidney biopsy	Clinical study	[96]
SLE	Human	Mut	Decreased	Peripheral blood leukocyte	Clinical study	[55]
SLE	Human	Mut	NA	Blood lymphocyte	Clinical study	[56]
Sjögren’s syndrome	Human	Decreased	Decreased	Leukocytes	Clinical study	[55]
Bacterial peritonitis	Human	NA	Increased	Peritoneal fluid	Clinical study	[21]
Pelvic inflammatory disease	Human	NA	Increased	Peritoneal fluid	Clinical study	[78]
Inflammatory infection by *E. coli* and *Burkholderia cenocepacia* bacteria	Mouse	Decreased	NA	Macrophage	In vitro	[47]
*Listeria monocytogenes* *Lactobacillus paracasei* TLR ligands	Mouse	Decreased	NA	DC	In vitro	[22]
α-Mannosidosis	Human/Mouse	Decreased	NA	Leukocytes or other nucleated cells	Clinical study	[3]
*Mycobacterium bovis*-infected bovine tuberculosis	Bovine	Increased	NA	Alveolar macrophage	Animal model	[45]
Tuberculosis infected with Mycobacterium	Human	Decreased	Decreased	Macrophage	In vitro	[46]
Influenza virus	Mouse	Decreased	NA	A549	In vitro	[48]
Porcine reproductive and respiratory syndrome virus (PRRSV) infection	*Sus scrofa*	Decreased	Decreased	Porcine alveolar macrophages Macrophage infected with virus	In vitro	[49]
Tumours							
Gynaecologic cancer	Human	NA	Increased	Peritoneal fluid Fluid from benign ovarian cysts	Clinical study	[78]
Glioma	Human	Increased	Increased	Tumour	In vitro	[20]
Glioma	Human	Increased	Increased	Tumour	Clinical study	[77]
Glioma	Human	Increased	Increased	Tumour	In vitro	[79]
BLCA/COAD/BRCA	Human	Increased	NA	Tumour	In vitro	[20]
Leukaemia	Human	Increased	Increased	Cancer cells	In vitro	[19]
Neurodegenerative disorders							
Alzheimer’s disease	Human	Increased	Increased	Fibroblast	Clinical study	[63]
DLB, AD and FTD	Human	NA	Decreased	Cerebrospinal fluid	Clinical study	[23]
Parkinson’s disease	Human	High	NA	Cerebrospinal fluid	Clinical study	[95]
	Human	Decreased	NA	Peripheral blood leukocytes	Clinical study	[62]
Progressive neurological symptoms and brain atrophy	Dog	Decreased	Decreased	Brain	Animal model	[68]
Others							
Irradiation	Mouse	Increased	NA	Liver	Animal model	[86]
Morpholine	Rabbit	NA	High	Alveolar macrophages	Animal model	[88]
Rapamycin	Mouse/Drosophila	Increased	NA	Gut	Animal model	[87]
Renal failure	Human	Decreased	Decreased	Pathologic examination	Clinical study	[82]
Pulmonary emphysema	Human	Decreased	NA	Lung	Clinical study	[85]

AD, alzheimer′s disease; BLCA, bladder urothelial carcinoma; BRCA, breast invasive carcinoma; COAD, colon adenocarcinoma; DLB, dementia with Lewy body; FTD, frontotemporal dementia; Mut, mutation; NA, not attention; SLE, systemic lupus erythematosus.

### Infectious diseases and inflammatory activation

The extracellular secretion of lysosomal enzymes holds significant relevance in initiating and sustaining inflammatory responses [42, 43]. Elevated α-mannosidase activity was detected in cell-free cerebrospinal fluid (CSF) obtained from patients diagnosed with bacterial meningitis, but not from those with aseptic meningitis [44]. This observation strongly suggests that inflammation could potentially induce heightened extracellular secretion of MAN2B1.

In a separate clinical investigation conducted by Nicholas G et al. [21], an assessment of lysosomal enzyme activity in the peritoneal fluid of patients with bacterial peritonitis, acute mesenteric lymphadenitis and control subjects devoid of peritoneal inflammation was conducted. The study revealed a substantial elevation in the median activity of α-mannosidase among patients with bacterial peritonitis, reaching a level 20 times higher than that observed in control subjects. Conversely, the enzyme activity in patients with acute mesenteric lymphadenitis did not significantly differ from that of the control group. Notably, no significant variance in α-mannosidase activity was observed between patients with peritonitis exhibiting negative bacterial cultures (under antibiotic treatment) and those with positive cultures. The detection of lysosomal mannosidase activity in peritoneal fluid stands as a valuable diagnostic tool for identifying patients afflicted with bacterial peritonitis. These results suggest the probable involvement of MAN2B1 specifically in bacterial-induced inflammation rather than aseptic inflammation. The observed escalation in mannosidase activity potentially correlates with bacterial recognition and phagocytic processes.

Several investigations have explored the association between MAN2B1 and bacterial infections. Notably, in studies pertaining to *Mycobacterium tuberculosis* infection, the alterations in MAN2B1 expression have led to conflicting findings. A study by Stephanie Widdison et al. [45] found heightened MAN2B1 expression in *Mycobacterium bovis*-infected bovine alveolar macrophages compared to those infected with *M. tuberculosis.* Interestingly, *M. bovis* consistently caused disease, whereas *M. tuberculosis* infection was effectively controlled. Conversely, Nathan J. Hare et al. [46] observed a divergent outcome in human macrophages infected with *M. tuberculosis.* Utilising LC–MS/MS and QPCR assays, they identified several N-glycosylation-modified enzymes, noting a four-fold decrease in MAN2B1 protein levels and a significant reduction in transcript levels 72 hours post-infection. Discrepancies between these outcomes could potentially be attributed to inter-species differences.

In an in vitro study conducted by Anna Torri et al. [22], dendritic cells (DCs) were subjected to various compounds, revealing intriguing insights. Stimulation of DC cells with bacterial strains such as *Listeria monocytogenes, Lactobacillus paracasei* and Toll-like receptor (TLR) ligands (LPS, poly I:C, ZymA) prompted inflammatory activation, concomitant with a noteworthy reduction in MAN2B1 mRNA expression. Interestingly, treatments involving vitD, IL-10, Nismesulide and IFNa failed to elicit changes in MAN2B1 expression. Similarly, Mohd M. Khan et al. [47] observed a reduction in MAN2B1 protein levels in macrophages 24 hours post-infection by *Escherichia coli* and *Burkholderia cenocepacia* bacteria. These collective findings suggest a nuanced relationship between MAN2B1 expression and the inflammatory response to specific bacterial stimuli.

Furthermore, studies have implicated MAN2B1 in the immune response against viral infections. In a genome-wide RNAi screen conducted by Alexander Karlas et al. [48], knockdown of MAN2B1 using siRNA inhibited the replication of influenza A/WSN/33 or A/Hamburg/04/2009 viruses in A549 cells, without affecting cell viability. Additionally, in Porcine Reproductive and Respiratory Syndrome Virus (PRRSV) infection of porcine alveolar macrophages, a cell type primarily targeted by the virus, MAN2B1 was among the genes showing significant downregulation [49].

**Figure 1.**
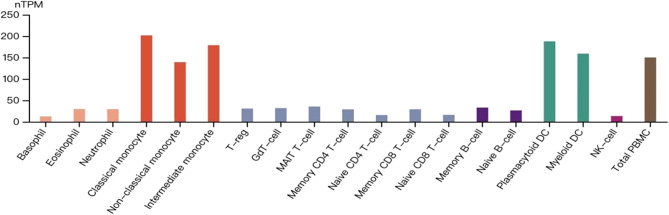
MAN2B1 expression across various immune cell types. The transcript expression values, computed as nTPM (normalised transcripts per million), were derived from an internal normalisation pipeline encompassing 18 distinct immune cell types and total peripheral blood mononuclear cells (PBMCs). Colour-coding is assigned according to the lineage of blood cell types. This depiction of blood cell-type expression provides an overview of RNA-seq data sourced from multiple references, including internally generated Human Protein Atlas (HPA) data, as well as datasets from studies by Monaco et al. [97] and Schmiedel et al. [98].

While existing research primarily concentrates on innate immune cells, there is a scarcity of reports on the atypical expression of MAN2B1 in adaptive immune cells. This paucity could potentially be linked to varying MAN2B1 expression levels across different cell types. To explore this, we analysed MAN2B1 mRNA levels across diverse immune cell types using data from the Human Protein Atlas (HPA) database (https://www.proteinatlas.org). Intriguingly, our analysis unveiled high expression levels of MAN2B1 in monocytes and DCs, whereas it exhibited lower expression in granulocytes, T cells, B cells and NK cells (Figure 1). These findings indicate a distinctive pattern of MAN2B1 expression across various immune cell types, possibly highlighting its varied role within the immune system.

### Autoimmune diseases

Protein glycosylation produces structural variation at the cell surface and contributes to immune self-recognition [4, 5]. Under normal circumstances, the immune system responds to foreign antigen epitopes [50]. However, autoimmunity arises when enzyme abnormalities cause overlapping glycosylation patterns between host and pathogen. This altered glycosylation leads to immune recognition of new glycosyl epitopes, often linked to various autoimmune syndromes [51]. Golgi α-Mannosidase II (αM-II), encoded by MAN2A1, localises to the Golgi apparatus and catalyses the final hydrolytic step in the asparagine-linked oligosaccharide (N-glycan) maturation pathway. Deficiency of αM-II has been linked to the development of a systemic lupus erythematosus (SLE)-like autoimmune disease in mice [52, 53].

Indications are pointing towards a potential association between MAN2B1 and autoimmune diseases. In a seminal report by Maki Urushihara in 2004 [54], two sisters diagnosed with α-mannosidosis at ages 11 and 14 later developed SLE at ages 23 and 25, respectively. Mutation analysis revealed a 2548C deletion in exon 21 of the LAMAN gene, leading to a novel termination codon 924. Similarly, Patryk Lipin ´ski [55] reported a case where a girl diagnosed with α-mannosidosis at 3 years old subsequently developed Sjögren’s syndrome at 7 and SLE at 9 years of age. Genetic analysis identified the mutation c.2245C>T, p. (Arg749Trp) in MAN2B1 in this case. Additionally, MAN2B1 was identified as one of the causative mutations in an atypical and severe SLE patient [56]. The case involved an eight-year-old boy from a consanguineous family who was newly diagnosed with SLE, revealing homozygous mutations in MAN2B1 and SLC7A7 (Solute Carrier Family 7 Member 7) through whole exome sequencing. These clinical cases emphasise a strong association between mutations in MAN2B1 and autoimmune diseases.

Collectively, these discoveries emphasise the intricate interplay between MAN2B1 and immunity. Infections, notably bacterial meningitis and peritonitis, displayed heightened lysosomal mannosidase activity, suggesting the involvement of MAN2B1 in bacterial-induced inflammation. Investigations into bacterial and viral stimuli revealed diverse MAN2B1 expression patterns, highlighting nuanced connections between MAN2B1 and specific pathogens. Notably, MAN2B1 exhibits elevated expression in monocytes and dendritic cells, pivotal in the body’s immune defence against infections and inflammatory responses. Clinical evidence strongly associates MAN2B1 mutations with autoimmune diseases such as SLE and Sjögren’s syndrome.

## MAN2B1 and neurodegenerative disorders

Lysosomal dysfunction and perturbation of lysosomal protein function are recognised features in various neurodegenerative diseases, including Alzheimer’s disease (AD), Parkinson’s disease (PD), Lewy body dementia (DLB) and anterior temporal dementia (FTD) [57-60]. It is well-documented that lysosomal dysfunction contributes to the pathogenesis of neurodegenerative diseases [61]. In a comprehensive study involving 536 patients with sporadic early-onset Parkinson’s disease (SEOPD) and 600 patients with familial PD across Asian populations, researchers conducted genetic assessments of 67 candidate lysosome-related genes using whole exome sequencing. Their findings indicated a significant enrichment of rare damaging variants in MAN2B1 associated with PD [62].

Carla Emiliani and colleagues reported an upregulation of α-D-mannosidase activity and transcript levels in skin fibroblasts of individuals with AD [63]. Conversely, Parnetti’s team investigated lysosomal α-D-mannosidase activity in the CSF of patients diagnosed with DLB, AD and FTD, along with healthy subjects. Their findings revealed significantly lower enzyme activity in the CSF of patients compared to healthy individuals [23]. In a subsequent study, researchers analysed α-mannosidase in venous blood and CSF from patients diagnosed with mild neurological disorders, utilising DEAE cellulose chromatography [64]. The results highlighted that α-mannosidase from venous blood did not penetrate the blood–brain barrier, while the enzyme activity detected in CSF originated from cerebral lysosomes. These differing outcomes could be attributed to the diversity in sample types examined. CSF, being more directly correlated with pathological changes in the CNS, contrasts with fibroblasts in the skin. The observed enzyme activity in CSF is believed to potentially reflect pathological cerebral changes associated with neurodegenerative diseases [64].

Progressive intellectual decline, motor dysfunction and cerebellar atrophy stand out as prominent neurological symptoms in individuals diagnosed with mannosidosis. Line Borgwardt’s research [13] explored genotype–phenotype associations in α-mannosidosis, revealing a robust correlation between MAN2B1 genotype, intracellular localisation and CNS symptoms. Specifically, this association extended to pulmonary function, upper limb coordination, balance, FVC% and the accumulation of oligosaccharides in CSF.

Extensive evidence from diverse animal models further underlines the link between α-mannosidosis and CNS abnormalities. Guinea pigs affected by α-mannosidosis exhibited significant neurological abnormalities as early as 2 months of age, marked by neuronal lysosomal vacuolation and secondary GM3 ganglioside accumulation. They also demonstrated extensive axonal globus pallidus and diminished white matter myelin sheaths [65]. In mice with α-mannosidosis, observable traits included activated Bergman glial populations, macrophage infiltration and the buildup of free cholesterol and gangliosides [66]. Notably, ERT exhibited partial reversal of cerebellar pathological changes, reducing activated macrophages and astrocytes while enhancing neurocognitive function [66, 67]. Recently, a seven-month-old dog displaying progressive neurological symptoms and brain atrophy was euthanised, and microscopic examination confirmed a lysosomal storage disease. Whole-genome sequencing unveiled a MAN2B1 missense mutation (Asp104Gly) with undetectable α-mannosidase activity [68].

LSDs and age-related neurodegenerative disorders such as PDs and ADs are characterised by a common pathological feature: the accumulation of undigested material within the organelles of the endolysosomal system [69, 70]. Moreover, members of the class II α-mannosidase family, namely MAN2A1 [71] and MAN2C1 [72], have also been reported to be associated with neurodegenerative conditions. This implies that the pathologic alterations in lysosomes related to neurodegenerative diseases may involve glycosylation beyond the sole influence of MAN2B1.

## MAN2B1 and cancers

Glycosylation stands as a hallmark of cancer, intricately involved in various aspects of cancer cell biology encompassing cell signalling, tumour cell dissociation, invasion, cell–matrix interactions, angiogenesis and immune modulation [73]. Eliminating N-glycan expression on tumour cells has shown promise in enhancing the efficacy of immunotherapy [74, 75]. Disruptions in glycosylation patterns can arise from irregularities in the expression, localisation and functioning of glycosyltransferases and glycosyl hydrolases [76].

Notably, abnormal upregulation of α-D-mannosidase has been linked to Ras activation in Alzheimer’s disease [63]. Ras signalling, pivotal in normal cell proliferation, frequently undergoes activation across a spectrum of malignancies. While the correlation between α-D-mannosidase and Ras activation remains unreported in cancers, upregulation of MAN2B1 and heightened α-mannosidase activity have been documented in various cancers, including gynaecologic cancer, malignant glial tumours and leukaemia [19, 77, 78]. Analysis of The Cancer Genome Atlas (TCGA) data unveiled elevated mRNA levels of MAN2B1 across numerous human cancers, encompassing bladder urothelial carcinoma (BLCA), breast invasive carcinoma (BRCA) and colon adenocarcinoma (COAD) [20].

N.G. Beratis [78] reported a notable elevation in α-mannosidase activity in the peritoneal fluid of patients with gynaecologic cancer compared to women with infertility, serving as control subjects, in a study conducted in 2004. However, no notable variance in α-mannosidase activity was detected in the fluid extracted from ovarian cysts, indicating its potential diagnostic significance in distinguishing malignant gynaecologic cancers. α-D-mannosidase activity surged by nearly 70-fold in the HL60 cell line and 35-fold in the NB4 promyelocytic leukaemia cell line compared to skin fibroblasts [19]. Gene expression analysis unveiled a close relationship between heightened enzyme activity and transcriptional upregulation. Notably, MAN2B1 transcriptional regulation differs between normal and tumour cell lines. In HEK-293 cells, Sp1 binds to the promoter region −101/−71 of MAN2B1, encompassing two overlapping GC boxes. Conversely, NF-κB regulates MAN2B1 transcription in the HL60 cell line by binding to the segment −373/−269.

In a 2006 study conducted by P. Wielgat [77], the expression of lysosomal exoglycosidases was examined in 29 specimens of human gliomas along with unchanged brain tissue typically excised alongside the tumours. The study revealed a significant increase in α-mannosidase activity within malignant glial tumours compared to both control tissue (normal brain tissue) and non-glial tumours. The most substantial rise in α-mannosidase activity was observed in anaplastic astrocytomas. Moreover, primary tumours exhibited markedly higher α-mannosidase activity than metastatic tumours. This study was the first to suggest a correlation between α-mannosidase activity and the progression of brain tumours, implying a potential role of lysosomal exoglycosidases in the advancement and dynamic development of glial tumours. Two recent RNA sequencing-based bioinformatic analytical studies have identified MAN2B1 as a novel prognostic biomarker for glioma, indicating that higher expression of MAN2B1 correlates with shorter survival times among patients [20, 79]. These studies highlighted a significant increase in MAN2B1 expression within glioma tissue, revealing associations with WHO classification, IDH1 mutation status and various histological subgroups among individuals diagnosed with glioma. Additionally, the research illustrated a positive connection between MAN2B1 and immune response pathways, coupled with infiltration of immune cells. Notably, MAN2B1 exhibited a strong positive correlation with M2 macrophage markers (TGFBI and CD163), while its relationship with M1 macrophage markers (NOS2 and TNF) appeared comparatively weaker.

These studies collectively suggest a potential link between heightened α-mannosidase activity and the advancement of malignant tumours. Furthermore, the correlation between MAN2B1 and tumour-associated macrophages indicates possible involvement of MAN2B1 in shaping the tumour microenvironment.

## MAN2B1 and others disorders

α-Mannosidosis, an inherited metabolic disorder, exhibits characteristic cellular changes such as multiple membrane-bound cytoplasmic vacuoles found in various cell types—hepatocytes, pancreatic exocrine cells, renal cells, thyroid cells, smooth muscle cells, bone cells and neurons in the CNS and peripheral nervous system [80]. Reduced α-mannosidase activity affects not only the nervous and immune systems but also involves other organs, leading to liver or spleen enlargement, lung issues [81] and kidney complications [82]. In a guinea pig model of α-mannosidosis, α-mannosidase deficiency triggers substantial morphological alterations in foetal pathology [83]. ERT exhibits potential in reducing lysosomal vacuolation in the liver, kidneys, spleen and pancreas [84]. MAN2B1 has been associated with pathological alterations in multiple organs, in addition to its role in α-mannosidosis.

In a multi-ethnic genome-wide association study encompassing 7,914 participants, Ani Manichaikul and colleagues [85] explored the association between lung/single nucleotide polymorphism (SNP) health. They identified the MAN2B1 SNP (rs10411619) in Hispanic patients and the MAN1C1 SNP (rs12130495) in African Americans. In an independent multi-ethnic cohort, they found that gene expression of MAN2B1 in peripheral monocytes showed a statistically significant association with a reduced upper-lower lobe ratio, whereas the gene expression of MAN1C1 was associated with an increased percentage of emphysema.

Lysosomal enzymes play pivotal roles in various biological processes and cellular reactions. Reports indicate that specific stimuli can induce alterations in MAN2B1 expression. For instance, Daila S. Gridley demonstrated that low-dose photons and solar particle events notably upregulated MAN2B1 expression in mouse liver [86]. In another study by Paula Juricic et al. [87], chronic rapamycin treatment in female Drosophila and mice elicited a geroprotective response. Rapamycin induced autophagy in the mouse intestine, as observed by an increased count of lysozyme^+^/p62^+^ granules per Paneth cell. Immunofluorescence data displayed an increased presence of MAN2B1^+^ punctae in intestinal crypts, persisting even six months post-treatment. This elevation in MAN2B1 expression potentially signifies alterations within cellular lysosomes, potentially serving as a marker for Rapamycin-induced autophagic activity and implicating MAN2B1 in intestinal homeostasis regulation post-Rapamycin treatment. Additionally, an experimental study on rabbits revealed that inhalation of morpholine vapour significantly augmented α-mannosidase activity in alveolar macrophages [88]. in vitro experiments further confirmed that exposing alveolar macrophages to morpholine induced a time-dependent elevation in α-mannosidase activity. These alterations in α-mannosidase activity may indicate the cellular response to the biological toxicity of certain substances.

## Conclusion and prospection

**Figure 2.**
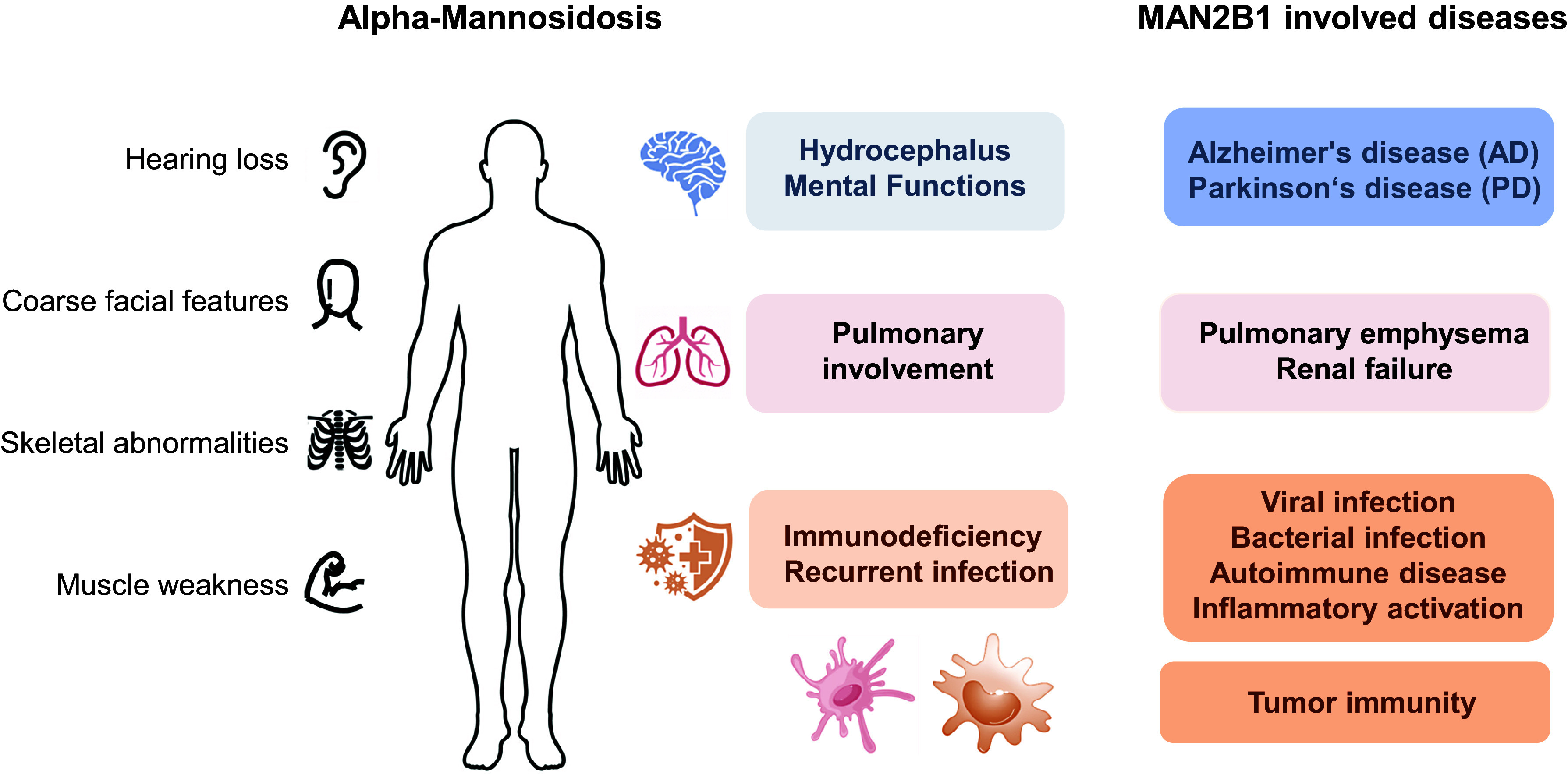
Clinical manifestations of α-mannosidosis and disorders associated with MAN2B1.

Investigations extending beyond α-mannosidosis have unveiled notable insights into the potential role of MAN2B1 in inflammatory conditions, particularly in macrophage infection and activation. These studies delineate variations in MAN2B1 expression and activity across various pathological contexts, shedding light on its associations with inflammation and tumorigenesis. However, comprehensive exploration through systematic experimental studies remains pending. Intriguingly, the involvement of MAN2B1 in infections, nervous system, autoimmune disorders and organ damage shows certain correlations with symptoms observed in α-mannosidosis (Figure 2). A comprehensive investigation into MAN2B1-related research is anticipated to significantly advance our understanding of the pathogenic mechanisms underlying α-mannosidosis, potentially opening avenues for more diverse and effective therapeutic strategies. However, we have no ability to draw definitive conclusions regarding the relationship between MAN2B1 and various disease processes now. The potential cellular and molecular mechanisms remain to be confirmed through further experimental validation. Consequently, there is a pressing need for a concerted effort to enhance the basic and clinical knowledge of MN2B1 for the treatment of these diseases.

Lysosomal changes and dysfunction have been identified in a wide variety of diseases, including autoimmune, metabolic and kidney diseases, PD, diabetes mellitus and lysosomal storage diseases [43, 89]. Targeting the lysosome has emerged as a promising approach in various therapeutic strategies, especially in treating LSD and some other diseases related to lysosomal dysfunction. However, challenges persist in achieving specific drug delivery, controlling lysosomal enzyme activity and ensuring effective disease impact. The complexity of lysosomal functions complicates the development of targeted therapies. Consequently, unravelling the molecular intricacies within lysosomes would offer more precise therapeutic targets for diseases associated with lysosomal dysfunction.

Aberrant glycosylation, considered a hallmark of cancer, has been implicated in its initiation and progression across various malignancies [90-92]. The transmembrane protein GManII, encoded by MAN2A1, catalyses the initial step in complex N-glycan biosynthesis. Inhibition of GManII in cancer cells impedes cancer progression and enhances chemosensitivity. MAN2A1 has emerged as a therapeutic target for cancer treatment. Swainsonine, the most potent GManII inhibitor, exhibiting nanomolar affinity with the GH 38 mannosidase family, has garnered attention as a potential anticancer agent [93]. However, challenges arise in targeting GManII due to the high similarity in functionality and active sites between LMan encoded by the MAN2B1 gene and GManII. Swainsonine, a reversible inhibitor, has shown promising results in preclinical and phase I trials but was discontinued in phase II due to disease progression or adverse reactions [94]. The adverse effects included mild symptoms like fatigue, anorexia, nausea and diarrhoea, while severe reactions were primarily associated with neurological toxicity such as depression, anxiety and cognitive impairment. Nonetheless, these adverse effects were fully reversible upon treatment cessation. Researchers are inclined to develop swainsonine analogues with improved selectivity to overcome these side effects. Similarly, developing small molecule drugs targeting MAN2B1 might face functional interference and side effects on MAN2A1. Hence, gene therapy or targeted delivery could be pivotal strategies in treating related disorders, especially targeting macrophages.
